# Avibirnavirus VP4 Protein Is a Phosphoprotein and Partially Contributes to the Cleavage of Intermediate Precursor VP4-VP3 Polyprotein

**DOI:** 10.1371/journal.pone.0128828

**Published:** 2015-06-05

**Authors:** Sanying Wang, Boli Hu, Weiying Si, Lu Jia, Xiaojuan Zheng, Jiyong Zhou

**Affiliations:** 1 Key Laboratory of Animal Virology of Ministry of Agriculture, Zhejiang University, Hangzhou, PR China; 2 College of Veterinary Medicine, Nanjing Agricultural University, Nanjing, PR China; 3 State Key Laboratory and Collaborative Innovation Center for Diagnosis and Treatment of Infectious Diseases, First Affiliated Hospital, Zhejiang University, Hangzhou, PR China; 4 Shaoxing Center for Disease Control and Prevention, Shaoxing, PR China; CSIC/CNB, SPAIN

## Abstract

Birnavirus-encoded viral protein 4 (VP4) utilizes a Ser/Lys catalytic dyad mechanism to process polyprotein. Here three phosphorylated amino acid residues Ser538, Tyr611 and Thr674 within the VP4 protein of the infectious bursal disease virus (IBDV), a member of the genus Avibirnavirus of the family Birnaviridae, were identified by mass spectrometry. Anti-VP4 monoclonal antibodies finely mapping to phosphorylated (p)Ser538 and the epitope motif ^530^PVVDGIL^536^ were generated and verified. Proteomic analysis showed that in IBDV-infected cells the VP4 was distributed mainly in the cytoskeletal fraction and existed with different isoelectric points and several phosphorylation modifications. Phosphorylation of VP4 did not influence the aggregation of VP4 molecules. The proteolytic activity analysis verified that the pTyr611 and pThr674 sites within VP4 are involved in the cleavage of viral intermediate precursor VP4-VP3. This study demonstrates that IBDV-encoded VP4 protein is a unique phosphoprotein and that phosphorylation of Tyr611 and Thr674 of VP4 affects its serine-protease activity.

## Introduction

Infectious bursal disease virus (IBDV), a member of the genus Avibirnavirus of the family Birnaviridae, damages the precursors of antibody-producing B lymphocytes in the bursa of Fabricius and causes severe immunosuppression and mortality in young chickens. The IBDV genome is characterized by a bisegmented double-stranded RNA (segments A and B). The smaller segment B only encodes the VP1 with a molecular weight of 90 kDa. VP1 is the putative RNA-dependent RNA polymerase which interacts with the viral genome [1, 2] and is involved in IBDV mRNA translation via association with the carboxy-terminal domain of the eukaryotic translation initiation factor 4AII [3]. It has also been demonstrated to affect viral replication kinetics and modulate the virulence [4–6]. The larger segment A contains two partially overlapping open reading frames (ORFs) [7]. The smaller ORF encodes the VP5 protein, a 17-kDa nonstructural protein which interacts with host proteins, subunit p85α of PI3K and voltage-dependent anion channel 2, and plays important roles in regulating virus release and apoptosis [8–10]. The larger ORF encodes a 110-kDa polyprotein precursor that can be cleaved by the proteolytic activity of VP4 into the precursor of VP2 (pVP2, 48 kDa), VP3 (32 kDa) and VP4 (28 kDa) [11]. During virion maturation, pVP2 is further processed into the mature capsid protein VP2 (41 kDa) and four small peptides [12–14]. VP2 carries the major immunogenic determinants [15, 16] and contributes significantly to apoptosis, cell tropism, virulence and pathogenicity of virulent IBDV [17–19]. VP3, a major immunogenic and scaffolding protein of IBDV [20, 21], was found to interact with VP1 [22] and bind to the viral dsRNA forming ribonucleoprotein complexes [23], as well as thought to be a key organizer in virion morphogenesis [21]. VP4, as the viral protease of Birnaviruses, has been proposed to utilize a Ser/Lys catalytic dyad mechanism to process the polyprotein [11, 24]. VP4 forms regular needle-like structures called type II tubules within the cytoplasm and nucleus of IBDV-infected cells [25]. Meanwhile, current research data shows that *E*.*coli*-expressed VP4 protein can self-assemble into functional tubule-like particles and its activity can be completely inhibited by 1 mM of Ni^2+^ ions [26].

Recently, more attention has been paid to the functions of viral protein phosphorylation during virus infection. Phosphorylation at the Ser224 site of ICP0 of herpes simplex virus type 1 is known to be required for efficient viral replication [27]. Phosphorylated sites at Ser479 and Ser510 of the N protein in measles virus are important for the activation of viral mRNA transcription and/or replication of the genome *in vivo* [28]. In hepatitis C virus, the phosphorylated site at Ser222 of NS5A functions as a negative regulator of RNA replication [29]. The phosphorylation of Ser60, Ser64, and Thr62 of the P protein of vesicular stomatitis virus is critical for viral genome RNA encapsidation and template function [30]. Dephosphorylation of VP40 at sites Tyr7, Tyr10, Tyr13 and Tyr19 of Marburg virus impairs its ability to recruit nucleocapsid structures into filopodia, causing release of virions with low infectivity [31]. Phosphorylation of the capsid protein of West Nile virus mediated by protein kinase C has been shown to enhance its binding to HDM2 protein and importin and subsequently induce p53-dependent apoptosis [32]. The protein kinase A-mediated phosphorylation of Vpr at Ser79 site was found to be crucial for cell cycle arrest in HIV infection [33]. All these examples illustrate that phosphorylation of viral proteins plays important roles in regulating processes such as gene expression, viral replication and cell cycle arrest during viral infection.

Other than being reported as a serine-protease and an intracellular tubule type II, the VP4 protein of IBDV was also found to have novel roles as a biomarker for discriminating between pathogenic and nonpathogenic IBDV infections [34] and an inhibitor suppressing the expression of type I interferon via interaction with the glucocorticoid-induced leucine zipper [35]. In our previous proteomic analysis of IBDV-infected cells, different protein spots of VP4 were evident in the two-dimensional electrophoresis (2-DE) gel [36]. As it was of interest to learn whether these spots represented post-translational modifications, in this study we identified the phosphorylation sites of VP4 and generated monoclonal antibodies (mAbs) against phospho- and nonphospho-VP4 protein. Additionally, an in-depth analysis of the protease activity of phospho-VP4 was conducted.

## Materials and Methods

### Cells, vectors, virus, antibodies, reagents and animals

DF-1 cells and human embryonic kidney HEK-293T cells obtained from the American Type Culture Collection (ATCC, Manassas, VA) were cultured in Dulbecco’s modified Eagle's medium supplemented with 10% fetal bovine serum (FBS, Gibco-BRL Life Technologies, Grand Island, NY). IBDV strain NB (1.0 × 10^7^ TCID_50_/0.1 ml) was stored in our lab [37]. Rabbit polyclonal antibody (pAb) to VP4, chicken anti-VP2 pAb, mouse anti-VP3 mAb, pCI-neo-IBDV-TNT-A, pCI-neo-IBDV-VP4 and pEGFP-IBDV-VP4 were generated in our lab (our unpublished reagents). Lipofectamine 2000, Alexa Fluor 488 and 555 Protein Labeling Kits were obtained from Invitrogen (Carlsbad, CA). The 2-DE reagents were all from Bio-Rad Laboratories (Hercules, CA). The Qproteome Cell Compartment Kit was purchased from Qiagen (Hilden, Germany) and TNT T7 Quick Coupled Transcription/Translation System was from Promega (Madison, WI), respectively. Seven-week-old specific-pathogen-free (SPF) BALB/c mice were purchased from the Shanghai Laboratory Animal Center, Chinese Academy of Sciences, Shanghai, China. The animal study proposal was approved by the Institutional Animal Care and Use Committee (IACUC) of Zhejiang University (permit umber: SYXK 2012–0178). All animal experimental procedures were performed in accordance with the Regulations for the Administration of Affairs Concerning Experimental Animals approved by the State Council of People’s Republic of China.

### Virus infection, in-gel tryptic digestion, LC-MS/MS and MS data analysis

DF-1 cells were infected with IBDV at the multiplicity of infection (MOI) of 1, harvested at 24 h post-infection by scraping and centrifuged at 8,000 × *g* for 5 min. The pellets were dissolved with an equal volume of 2-DE lysis buffer containing 7 M urea, 2 M thiourea, 4% (wt/vol) CHAPS, 65 mM DTT, 0.2% Biolyte 3/10 and 1 mM phenylmethylsulfonyl fluoride. The lysates were subjected to 12% SDS-PAGE and immunoblot using a rabbit anti-VP4 pAb. Subsequently, the protein bands were manually excised from gels stained with colloidal Coomassie blue, and the in-gel tryptic digestion, LC-MS/MS and MS data analysis were performed as our previously reported methods [36, 38, 39].

### Generation and fine mapping epitopes of mAbs against IBDV VP4 protein

IBDV-infected cell lysates were separated by 12% SDS-PAGE, and the VP4 specific protein band was purified by eluting the protein from the excised gel in a dialyzer (Serva, Heidelberg, Germany) by electrophoresis in protein electrophoresis buffer (25 mM Tris base, 192 mM glycine, 3.5 mM SDS). The purified VP4 preparation was used as an immunogen and injected intraperitoneally into SPF BALB/c mice in order to generate mAbs to VP4 of IBDV as described previously [40, 41]. Reactivities of anti-VP4 mAbs were screened by an indirect immunofluorescence assay (IFA) and Western blot analysis. The phosphorylated and dephosphorylated antigenic epitope peptides of IBDV-VP4 (Table 1) were designed using three on-line prediction software programs (http://www.cbs.dtu.dk/services/BepiPred/, www.epitope-informatics.com/Links.htm, http://www.imtech.res.in/raghava/cbtope/submit.php) and synthesized using a Symphony Multiplex Peptide Synthesizer (Protein Technologies, Inc., Tucson, AZ). Peptide ELISA and peptide dot-ELISA were performed to test the reactivities of the mAbs with peptides as described previously [40]. After an immunoactive peptide was identified, its N-truncated, C-truncated and Ala-substituted derivatives were further synthesized and used to define the epitope motif by ELISA.

**Table 1:** Synthetic peptides of the IBDV VP4 protein in this study.

Peptide name	Amino acid sequence
Pep533-549pSer538	533cDGILAS*PGVLRGAHNLD549
Pep533-549	533cDGILASPGVLRGAHNLD549
Pep602-619pTyr611	602cTLSGHRVYGY*APGGVLP619
Pep602-619	602cTLSGHRVYGYAPGGVLP619
Pep667-683Thr674	667cVPIHVAMT*GALNA683
Pep667-683	667cVPIHVAMTGALNA683
Pep515-558	515cKGYEVVANLFQVPQNPVVDGILASPGVLRGAHNLDCVLREGATL558
Pep550-565	550CVLREGATLFPVVITT565
Pep563-600	563cITTVEDAMTPKALNSKMFAVIEGVREDLQPPSQRGSF600
Pep598-631	598cSFIRTLSGHRVYGYAPGGVLPLETGRDYTVVPID631
Pep618-641	618cPLETGRDYTVVPIDDVWDDSIMLS641
Pep638-662	638cIMLSKDPIPPIVGNSGNLAIAYMDV662
Pep653-674	653cGNLAIAYMDVFRPKVPIHVAMT674
Pep680-706	680CGEIEKVSFRSTKLATAHRLGLKLAGP706
Pep700-717	700cGLKLAGPGAFDVNTGPNW717
Pep713-741	713cTGPNWATFIKRFPHNPRDWDRLPYLNLPY741
Pep720-750	720cFIKRFPHNPRDWDRLPYLNLPYLPPNAGRQY750
Pep515-532	515cKGYEVVANLFQVPQNPVV532
Pep524-558	524cFQVPQNPVVDGILASPGVLRGAHNLDCVLREGATL558
Pep533-558	533cDGILASPGVLRGAHNLDCVLREGATL558
Pep524-539	524cFQVPQNPVVDGILASP539
Pep534-549	534cGILASPGVLRGAHNLD549
Pep543-558	543cRGAHNLDCVLREGATL558
Pep534-542	534cGILASPGVL542
Pep524-534	524cFQVPQNPVVDG534
Pep524-528	524cFQVPQ528
Pep529-534	529cNPVVDG534
Pep526-532	526cVPQNPVV532
Pep530-536	530cPVVDGIL536
Pep531-535	531cVVDGI535
Pep531-536	531cVVDGIL536
Pep532-536	532cVDGIL536
Pep533-536	533cDGIL536
Pep530-535	530cPVVDGI535
Pep530-534	530cPVVDG534
Pep530-536P530A	530cAVVDGIL536
Pep530-536V531A	530cPAVDGIL536
Pep530-536V532A	530cPVADGIL536
Pep530-536D533A	530cPVVAGIL536
Pep530-536G534A	530cPVVDAIL536
Pep530-536I535A	530cPVVDGAL536
Pep530-536L536A	530cPVVDGIA536

Note:

“*” indicates the amino acids with phosphorylation.

The letter “c” means cysteine appended to the Sulfo-SMCC cross-linker. Mutated residues are underlined.

### Subcellular and 2-DE analysis of VP4 molecules within IBDV-infected cells

Subcellular fractionation was performed using the Qproteome Cell Compartment Kit according to the manufacturer's protocol. Then the membrane, cytoplasmic, nuclear and cytoskeletal fractions obtained were subjected to SDS-PAGE, 2-DE and Western blot analysis with mouse mAb to the phospho-VP4 (P538-VP4) or nonphospho-VP4 (P530-VP4). Mock-infected cells were used as a negative control. Each reaction was performed in triplicate.

### Immunofluorescence staining

The indirect immunofluorescence assay was performed as described previously [36]. DF-1 cells or 293T cells were seeded in 96-well plates (Corning, New York, NY) or 35-mm glass bottom dishes (Shengyou Biotechnology, China), infected with IBDV or transfected with wild-type A segment, with wild-type VP4 or Ala/Asp VP4 mutants. The mouse mAb to phospho-VP4 or nonphospho-VP4 was used as primary antibody. The direct immunofluorescence assay was performed with Alexa Fluor 555 or Alexa Fluor 488 labeled mAbs to phospho-VP4 and nonphospho-VP4 on IBDV-infected DF-1 cells. Mock cells were used as negative controls. The nucleus was stained with 4’,6-diamidino-2-phenylindole (DAPI, Sigma). The stained cells were washed three times with PBST and subsequently examined under a Zeiss LSM510 laser confocal microscope.

### Site-directed mutagenesis and *in vivo* transfection

Various plasmids were generated using the pCI-neo-VP4 or pEGFP-VP4 plasmid as a template and PCR primers shown in Table 2 for site-directed mutagenesis. The PCR for dephospho- or phospho-mimicking recombinant VP4 was performed in a final volume of 25 μl containing 2.5 μl 10× Pyrobest buffer II (5 U/μl), 0.5 μl dNTP mixture (10 mM), 1 μl primers, 15 ng DNA template and 0.25 μl Pyrobest DNA polymerase, with the following conditions: denaturation at 94°C for 4 min, followed by 20 cycles of denaturation at 94°C for 45 s, annealing at 55°C for 45 s and extension at 72°C for 6 min, with a final elongation step at 72°C for 10 min. The PCR products were digested with *Dpn* Ι at 37°C for 1 h and confirmed by enzyme digestion and DNA sequencing. DF-1 cells were transfected with these recombinant plasmids using Lipofectamine 2000. At 6 and 15 h post-transfection, the cells were observed directly under the fluorescent microscope (pEGFP-transfected) or immunostained with anti-VP4 mAbs followed by FITC-conjugated secondary antibodies (pCI-neo-transfected).

**Table 2:** The summary of the primers used in this study.

Primer name	Nucleotide sequence	Length(bp)	location(nt)	Original
S538A-F	ACGGGATTCTTGCT***G***CACCTGGGGTACTC	29	1728–1756	T
S538A-R	GAGTACCCCAGGTG***C***AGCAAGAATCCCGT	29		
Y611A-F	CACAGAGTCTATGGA***GC***TGCTCCAGGTGGGGT	32	1946–1978	TA
Y611A-R	ACCCCACCTGGAGCA***GC***TCCATAGACTCTGTG	32		
T674A-F	TCCATGTGGCTATG***G***CGGGAGCCCTCAAT	29	2136–2164	A
T674A-R	ATTGAGGGCTCCCG***C***CATAGCCACATGGA	29		
S538D-F	ACGGGATTCTTGCT***GAC***CCTGGGGTACTC	29	1728–1756	TCA
S538D-R	GAGTACCCCAGG***GTC***AGCAAGAATCCCGT	29		
Y611D-F	CACAGAGTCTATGGA***G***A***C***GCTCCAGGTGGGGT	32	1946–1978	T T
Y611D-R	ACCCCACCTGGAGC***G***T***C***TCCATAGACTCTGTG	32		
T674D-F	TCCATGTGGCTATG***GAC***GGAGCCCTCAAT	29	2136–2164	ACG
T674D-R	ATTGAGGGCTCC***GTC***CATAGCCACATGGA	29		

Note: Italic and bold indicate mutation base.

### *In vivo* and *in vitro* proteolytic activity assay

Dephospho- or phospho-mimicking segment A with or without single and multiple mutations within VP4 were generated using pCI-neo-TNT-A as a template (unpublished data) following the same procedure as mentioned above. For the *in vivo* assessment of protease activity, 293T cells were transfected with various purified recombinant plasmids individually using Lipofectamine 2000 for 24 h. The cells were rinsed with PBS and lysed with RIPA buffer (50 mM Tris, 150 mM NaCl, 0.1% SDS, 1% TX-100, 0.5% sodium deoxycholate, 50 mM NaF and 0.2 mM Na_3_VO_4_). The supernatant was subjected to 12% SDS-PAGE and Western blot analysis to assess the cleavage activity of mutant segment A using the VP3 and nonphospho-VP4 (P530) mAbs.

The purified recombinant plasmids were further used to test the expression of polyprotein VP2/4/3 using the TNT T7 Quick Coupled Transcription/Translation System according to the manufacturer's instructions. Briefly, 40 μl T7 Quick master mix, 1 μl methionine (1 mM), 7 μl nuclease-free water and 2 μl plasmid (500 ng/μl) were incubated at 30°C for 90 min. The resultant samples were subjected to 12% SDS-PAGE and Western blot analysis to assess the cleavage activity of mutant segment A using the anti-VP3 and nonphospho-VP4 (P530) mAbs.

### Co-immunoprecipitation (Co-IP) assay

Co-IP experiments were performed as previous described [39]. Briefly, IBDV-infected and mock-infected cells were lysed with 500 μl NP-40 lysis buffer at 4°C for 30 min. Cell lysates were clarified by centrifugation at 8,000 × *g* for 10 min, and the supernatants were diluted with 500 μl PBS. The anti-phospho-VP4 mAb or anti-P530-VP4 mAb was added to the supernatants and incubated at 4°C for 8 h, and protein-A/G plus beads (Santa Cruz Biotechnology, Santa Cruz, CA) were added to the mixtures and incubated at 4°C for 8 h. Subsequently, the beads were washed with PBS three times and boiled with loading buffer, and the supernatants were prepared for SDS-PAGE and Western blot analysis.

## Results

### MS/MS identification of phosphorylated amino acid residues within VP4 protein

To identify whether the VP4 protein of IBDV is phosphorylated, IBDV-infected DF-1 cells were treated with 2-DE lysis buffer and separated by SDS-PAGE. Protein bands were excised manually from gels and sequentially subjected to in-gel digestion and MS identification by LC-MS/MS. As shown in Table 3, three phosphorylated peptides with the putative phosphorylation sites at Ser538 (S538), Tyr611 (Y611) and Thr674 (T674) were identified in VP4 protein of IBDV, indicating that the IBDV-encoded VP4 protein is a multi-site phosphorylated protein.

**Table 3:** LC-MS/MS identification of phosphorylation sites within VP4 of IBDV.

PepCount		UniquePepCount	CoverPercent	MW	PI	Identified Name					
391		22	27.37%	109685.64	5.98	gi|171906503|gb|ACB56951.1| polyprotein [IBDV]					
Scan(s)	Sequence	MH+	Diff(MH+)	Charge	Rank	XC	DeltaCn	Sp	RSp	Ions	PI
6558	573K.ALNSKMFAVIEGVR.E588	1535.8367	0.2217	2	1	3.9579	0.7645	1253.4	1	19|26	8.79
7263	642K.DPIPPIVGNSGNLAIAYM@DVFR.P665	2376.7167	0.1577	2	1	3.4656	0.5131	535.4	1	23|42	4.21
7892	642K.DPIPPIVGNSGNLAIAYMDVFR.P665	2360.7173	-1.0437	2	1	2.3315	0.4892	246.9	1	18|42	4.21
**8056**	**515K.GYEVVANLFQVPQNPVVDGILAS** ^**#**^ **PGVLR.G544**	**3033.3642**	**0.5692**	**3**	**1**	**4.2955**	**0.1037**	**971.1**	**1**	**37|108**	**4.37**
9345	515K.GYEVVANLFQVPQNPVVDGILASPGVLR.G544	2953.3843	1.4493	3	1	3.9103	0.1254	763.5	1	30|108	4.37
6954	702K.LAGPGVFDVNTGPNWATFIK.R723	2105.3808	-1.1372	2	1	4.29	0.6705	1043.2	1	21|38	5.84
4118	578K.M@FAVIEGVR.E588	1038.246	-0.814	2	1	3.2467	0.7753	1288.8	1	15|16	5.75
4887	578K.MFAVIEGVR.E588	1022.2466	-2.7244	2	1	3.3994	0.679	1476.9	1	15|16	5.75
1201	722K.RFPHNPR.D730	924.0445	-1.1675	2	1	2.48	0.7917	692.8	1	11|12	12
**4075**	**666K.VPIHVAM** @ **T** ^**#**^ **GALNACGGIEK.V686**	**2035.242**	**-0.067**	**2**	**1**	**2.2998**	**0.4792**	**310.6**	**1**	**15|36**	**6.71**
**3864**	**666K.VPIHVAMT** ^**#**^ **GALNACGGIEK.V686**	**2019.2426**	**1.7816**	**2**	**1**	**2.843**	**0.3507**	**633.6**	**1**	**17|36**	**6.71**
6467	623R.DYTVVPIDDVWDDSIM@LSK.D643	2228.4613	-1.0307	2	1	4.2199	0.8188	582.6	1	23|36	3.66
7372	623R.DYTVVPIDDVWDDSIMLSK.D643	2212.4619	-0.9071	2	1	4.164	0.2035	593.4	1	22|36	3.66
1740	587R.EDLQPPSQR.G597	1070.1383	-2.7667	2	1	2.2796	0.2897	541.9	1	13|16	4.37
6540	553R.EGATLFPVVITTVEDAM@TPK.A574	2136.4516	-0.4644	2	1	4.2235	0.7331	625.4	1	19|38	4.14
8591	553R.EGATLFPVVITTVEDAMTPK.A574	2120.4522	-1.1038	2	1	5.1746	0.8356	826.6	1	24|38	4.14
2941	543R.GAHNLDCVLR.E554	1155.283	0.674	1	1	2.6579	0.1734	440.5	1	12|18	6.74
6395	733R.LPYLNLPYLPPNAGR.Q749	1698.989	-0.183	1	1	3.252	0.6433	350.7	1	17|28	8.59
**5064**	**607R.VYGY** ^**#**^ **APDGVLPLETGR.D624**	**1787.88771**	**0.40771**	**2**	**1**	**2.5046**	**0.1962**	**276.5**	**1**	**16|45**	**4.37**
5613	607R.VYGYAPDGVLPLETGR.D624	1707.90781	-0.42919	2	1	4.3574	0.7967	1195.1	1	23|30	4.37

Note: The bold rows revealed the identified peptides that contained a phosphorylated amino acid.

^“#”^ is the phosphorylated amino acid residues.

^“@”^ indicates methylation of Methionine (M).

All proteins listed in the table were found to have a statistically significant p-value of less than 0.05.

### Generation and specificity of mAbs to VP4 protein of IBDV

**Fig 1:**
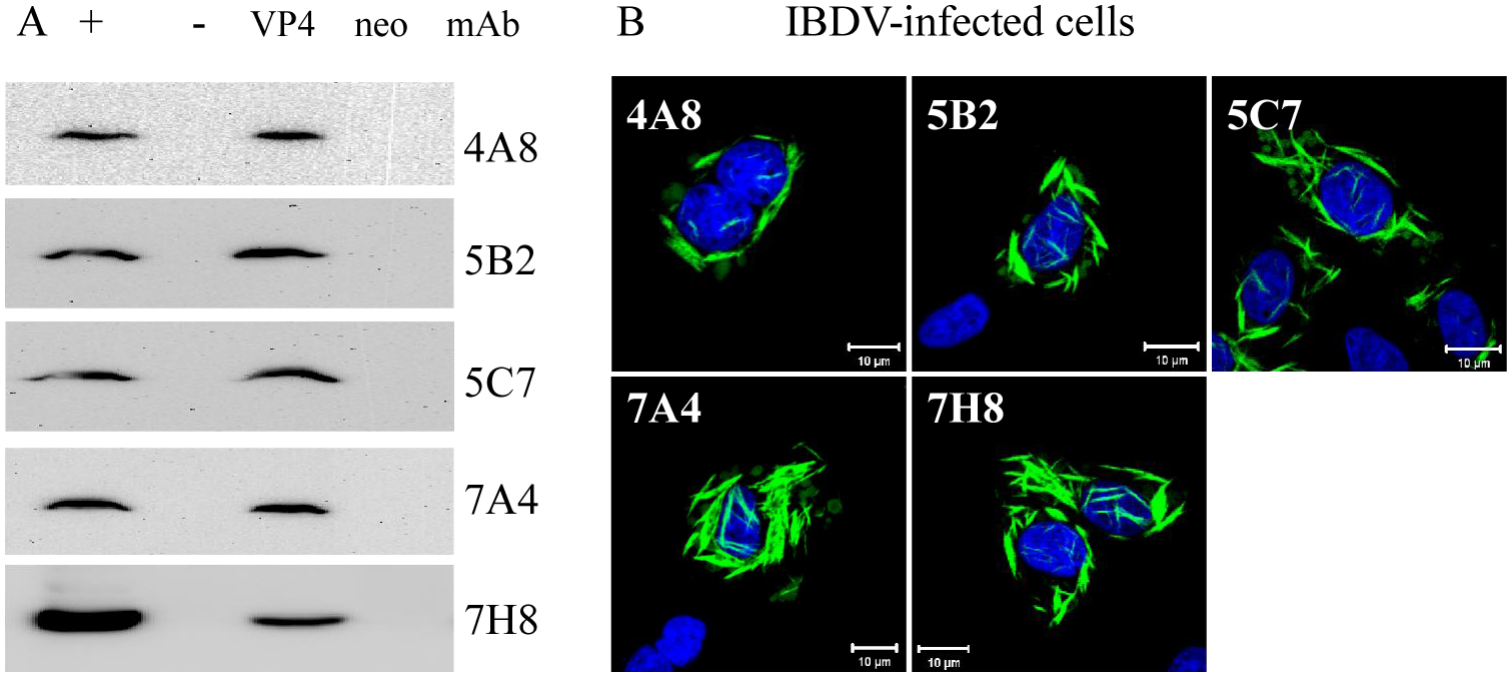
Reactivity and specificity of mAbs to IBDV VP4. (A) Western blot analysis of DF-1 cells infected with IBDV or transfected with pCI-VP4 for 24 h. The cells were lysed with NP-40 buffer and subjected to SDS-PAGE and Western blot analysis. The generated mAbs could react with the viral VP4 protein expressed in pCI-VP4 transfected cells and IBDV-infected cells. “+”: IBDV-infected cells; “-”: mock-infected cells; “VP4”: cells transfected with the recombinant vector pCI-neo-VP4; “neo”: cells transfected with the recombinant vector pCI-neo. (B) Immunofluorescence assay of DF-1 cells infected with IBDV and probed with mouse anti-VP4 mAb followed by FITC-conjugated goat anti-mouse. Nuclei were counterstained with DAPI.

The VP4 protein generated in IBDV-infected DF-1 cells was confirmed by Western blot analysis using the anti-VP4 pAb (S1 Fig), and then the specific protein band was gel-purified after separation by 12% SDS-PAGE. To prepare the mAb recognizing IBDV VP4, BALB/c mice were immunized with VP4 as the antigen. Ultimately, five hybridoma cell lines (4A8, 5B2, 5C7, 7A4 and 7H8) secreting mAbs to the VP4 of IBDV were cloned. Western blot analysis showed that these generated mAbs could specifically react with VP4 protein expressed in both VP4-transfected and IBDV-infected DF-1 cells (Fig 1A). IFA also indicated that these mAbs could recognize the VP4 protein in IBDV-infected cells (Fig 1B). However, by Western blot and IFA, these mAbs did not react with the viral proteins VP1,VP2, VP3 and VP5 of IBDV expressed in the transfected or infected cells (data not shown), confirming that these mAbs are specific for IBDV VP4 protein.

### Fine-mapping of linear antigenic epitope on VP4 protein of IBDV

**Fig 2:**
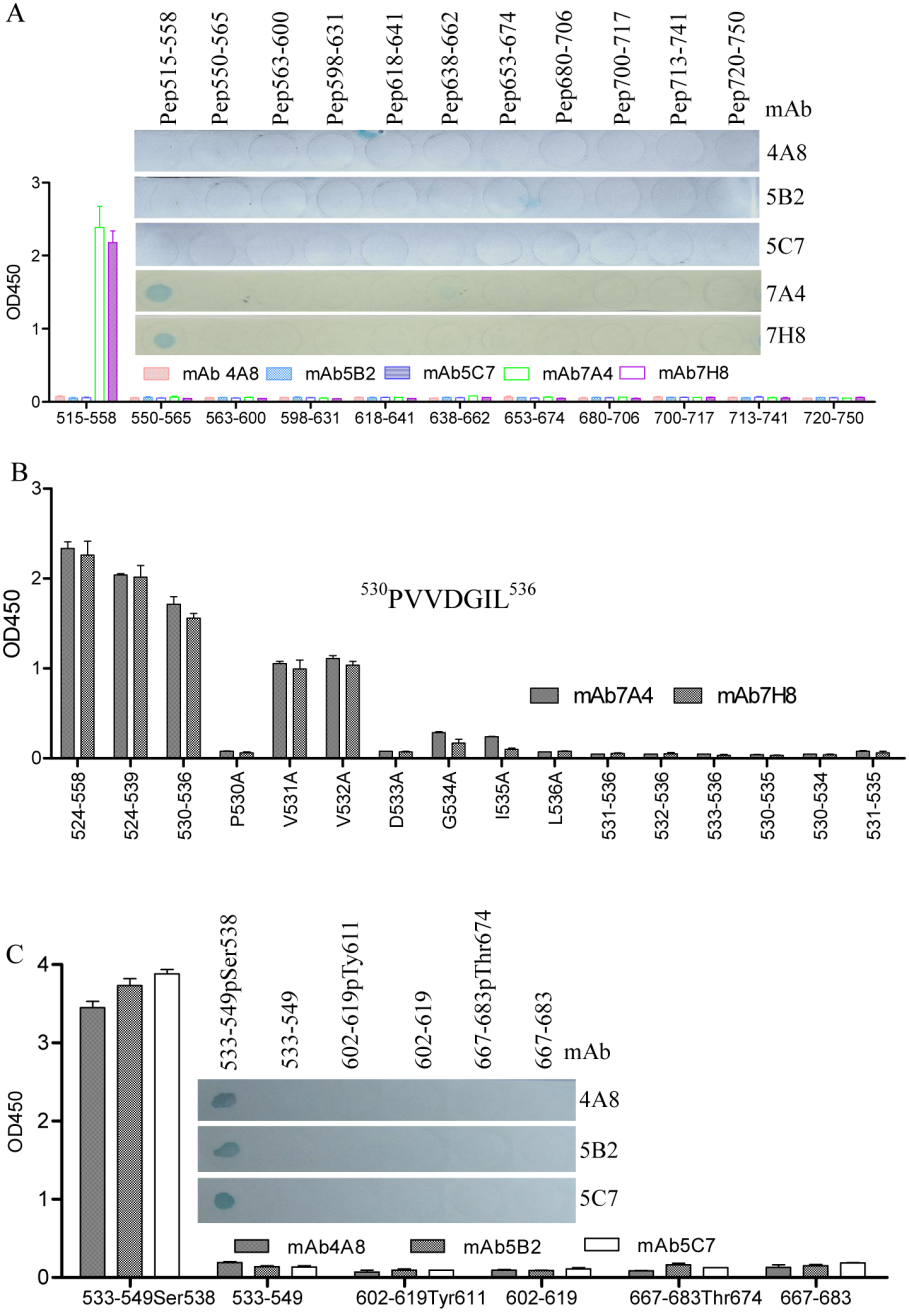
Fine mapping of epitopes on IBDV VP4 protein with peptide ELISA and peptide dot-ELISA. (A) Eleven BSA-conjugated peptides (spanning residues 515–558, 550–565, 563–600, 598–631, 618–641, 638–662, 653–674, 680–706, 700–717, 713–741 and 720–750) were coated on a 96-well plate in the peptide ELISA or dotted on a nitrocellulose membrane in the peptide dot-ELISA and probed with mAbs 4A8, 5B2, 5C7, 7A4 and 7H8 to the IBDV VP4 protein. The mAbs 7A4 and 7H8 could react with Pep515-558 but not the other peptides, and the mAbs 4A8, 5B2 and 5C7 did not react with any of these peptides. (B) The epitope motif of 7A4 and 7H8 was localized within the residues 530–536 by the peptide ELISA, and residues of ^530^Pro and ^533^DGIL^536^ were indispensable for forming the antigenic epitope. (C) BSA-conjugated phosphorylated and unphosphorylated peptides spanning residues 533-549pSer538, 533–549, 602-619pTyr611, 602–619, 667-683pThr674, 667–683 were coated on a 96-well plate in the peptide ELISA or dotted on a nitrocellulose membrane in a peptide dot-ELISA and probed with mAbs 4A8, 5B2 and 5C7 to the IBDV VP4 protein. The mAbs 4A8, 5B2 and 5C7 could recognize specifically the same phosphorylated peptide 533-549pSer538 but not unphosphorylated peptide 533–549.

Based on the LC-MS/MS identification data, the antigenic epitopes recognized by anti-VP4 mAbs were finely analyzed with a series of overlapping and phosphorylated linear peptides synthesized by the PEPSCAN technique (Table 1). Of the five mAbs tested in the peptide ELISA and peptide dot-ELISA, the mAbs 7A4 and 7H8 could react with the unphosphorylated peptide Pep515-558, while none of the synthesized peptides reacted with the mAbs 4A8, 5B2 and 5C7 (Fig 2A). Subsequently, in N- and C-terminally truncated peptides derived from unphosphorylated Pep515-558, the mAbs 7A4 and 7H8 could still react with the unphosphorylated peptide Pep530-536 (Fig 2B), indicating that the anti-VP4 mAbs 7A4 and 7H8 recognized the same linear B-cell epitope with the amino acid motif ^530^PVVDGIL^536^ (P530). The substitution analysis further revealed that the residues ^531^VV^532^ were dispensable for the antigenicity of the epitope, but any change of the residues of ^530^P and ^533^DGIL^536^ resulted in the loss of reactivity of the mAb (Fig 2B). These results indicated that ^530^P and ^533^DGIL^536^ are the crucial residues of the P530 epitope (P530 mAb). Interestingly, as shown in Fig 2C, the mAbs 4A8, 5B2 and 5C7 could react only with the phosphorylated Pep^533-549^Ser538 (pSer538), but not the phosphorylated Pep^602-619^Tyr611 (pTyr611) and Pep^667-683^Thr674 (pThr674) as well as unphosphorylated Pep^533-549^Ser538, Pep^602-619^Tyr611 and Pep^667-683^Thr674. These data demonstrated that the mAbs 4A8, 5B2 and 5C7 could recognize specifically the same phosphorylated epitope with phosphorylation at site Ser538 within the VP4 protein of IBDV (pSer538 mAb).

### Phosphorylated VP4 exists in complexes with different isoelectric points

**Fig 3:**
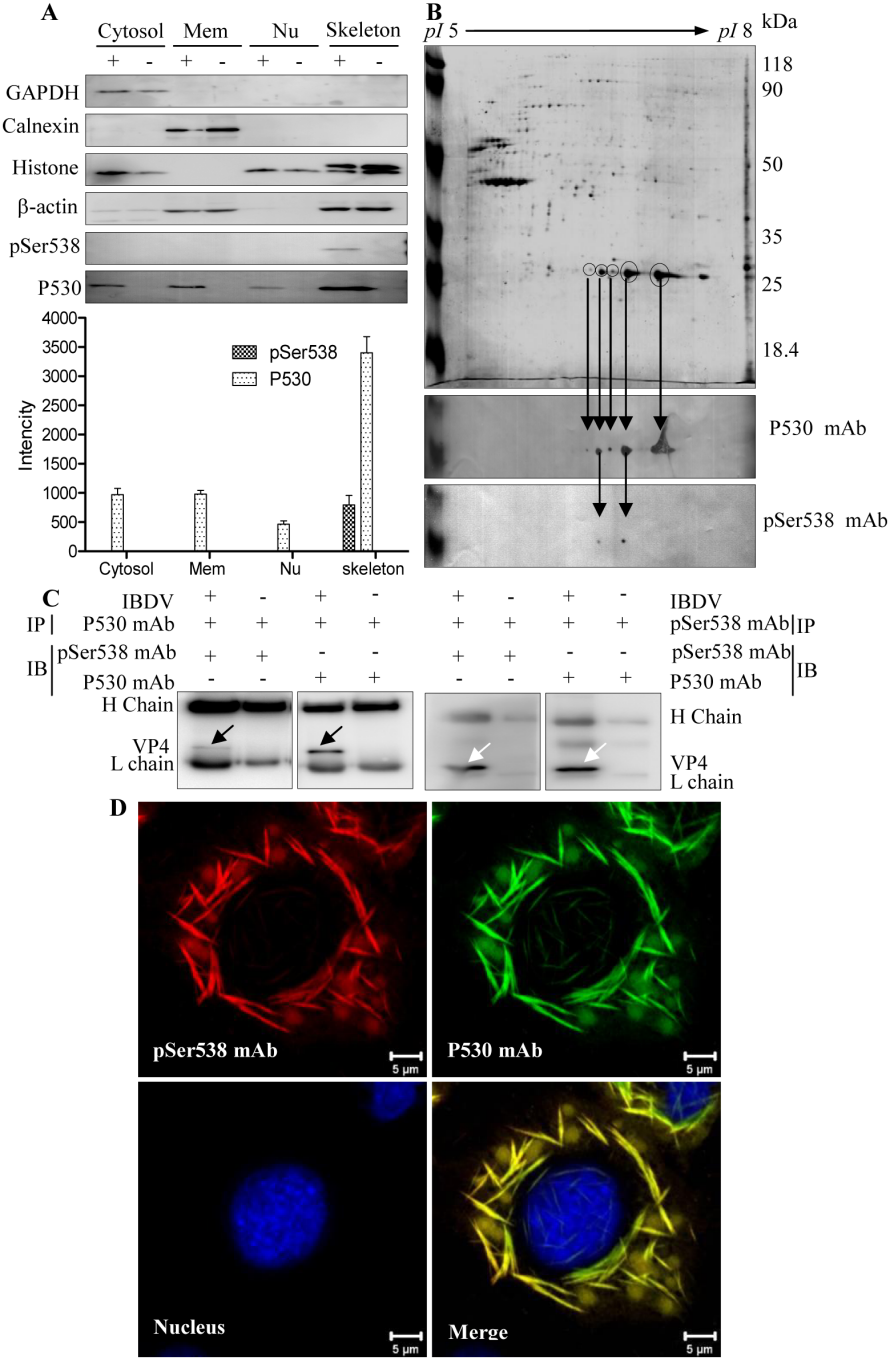
VP4 is a complex with different isoelectric points in IBDV-infected cells. (A) Western blot analysis of subcellular fractionated IBDV-infected DF-1 cells. The cells were infected with IBDV at the MOI of 1, harvested at 24 h post-infection and centrifuged at 8,000 × *g* for 5 min. The cytosolic, membrane, nuclear and cytoskeletal fractions of IBDV-infected (+) or mock-infected (-) DF-1 cells were sequentially isolated using the Qproteome Cell Compartment Kit. Equivalent amounts of each fraction (20 μg) were subjected to 12% SDS-PAGE followed by Western blot analysis. GAPDH, calnexin, histone H3 and β-actin were used as markers of the cytosolic, cell membrane, nuclear and cytoskeletal fractions, respectively. VP4 was detected with the anti-pSer538 or P530 mAb. Averaged densitometric intensities of three replicate immunoblots are shown. P530: VP4 protein recognized with the P530 mAb; pSer538: VP4 protein reacted with the pSer538 mAb. Error bar represents the standard deviation. (B) 2-DE and 2-DE blot analysis. Upper panel: Protein (200 μg) from the cytoskeletal fraction of IBDV-infected DF-1 cells were separated by 2-DE and visualized by colloidal Coomassie blue staining. Middle panel: Five VP4 protein spots recognized by the P530 mAb in a 2-DE blot. Lower panel: Two phosphorylated VP4 protein spots reacted with the pSer538 mAb in a 2-DE blot. (C) VP4 protein in IBDV-infected DF-1 cells was immunoprecipitated and detected with the P530 and pSer538 mAbs, respectively. (D) Co-localization of phosphorylated and unphosphorylated VP4 protein. Direct immunofluorescence assay of VP4 protein in IBDV-infected DF-1 cells with pSer538 and P530 mAbs. DF-1 cells were infected with IBDV, and fixed cells were probed with Alexa Fluor 555-labeled pSer538 (red) and Alexa Fluor 488-labeled P530 (green) mAbs. Nuclei were counterstained with DAPI. Overlapping signals were revealed by detection of VP4 by the P530 and pSer538 mAbs (merge).

To analyze the intracellular VP4 with and without the Ser538 phosphorylation, IBDV-infected DF-1 cells were separated into cell membrane, cytosol, nuclear and cytoskeletal fractions. As shown in the Western blot analysis of IBDV-infected cells (Fig 3A), the VP4 protein recognized by the P530 mAb (P530-VP4) was observed in all four fractions and mainly found in the cytoskeletal fraction. Meanwhile, the pSer538 mAb recognized VP4 protein (pSer538-VP4) accounted for a lower proportion than the P530-VP4 and was only detected in the cytoskeleton fraction and not in the cytosol, membrane and nucleus. For the cytoskeleton fraction of IBDV-infected cells, analysis by 2-DE and the corresponding blot revealed five MS/MS-identified and P530 mAb-recognized VP4 protein spots with different isoelectric points (Fig 3B Upper and Middle panels). Meanwhile, two of five protein spots could be recognized with the pSer538 mAb (Fig 3B Lower panel), and the pSer538-VP4 protein represented a lower proportion than that of the P530-VP4 protein. Additionally, Co-IP analysis demonstrated that the pSer538 mAb-recognized VP4 molecule also could be bound by the P530 mAb (Fig 3C), while the pSer538 mAb-recognized VP4 protein co-localized with the P530 mAb-reacted VP4 protein (Fig 3D). These results indicated that VP4 contains both the pSer538 and P530 epitopes (Fig 3C and 3D). Generally, the results demonstrated that IBDV VP4 was mainly detected within the insoluble cytoskeletal fraction, and pSer538-VP4 protein exists in complexes with different isoelectric points and is a minor protein in comparison with the P530-VP4.

### Phosphorylation modification is unrelated to intracellular accumulation of VP4

**Fig 4:**
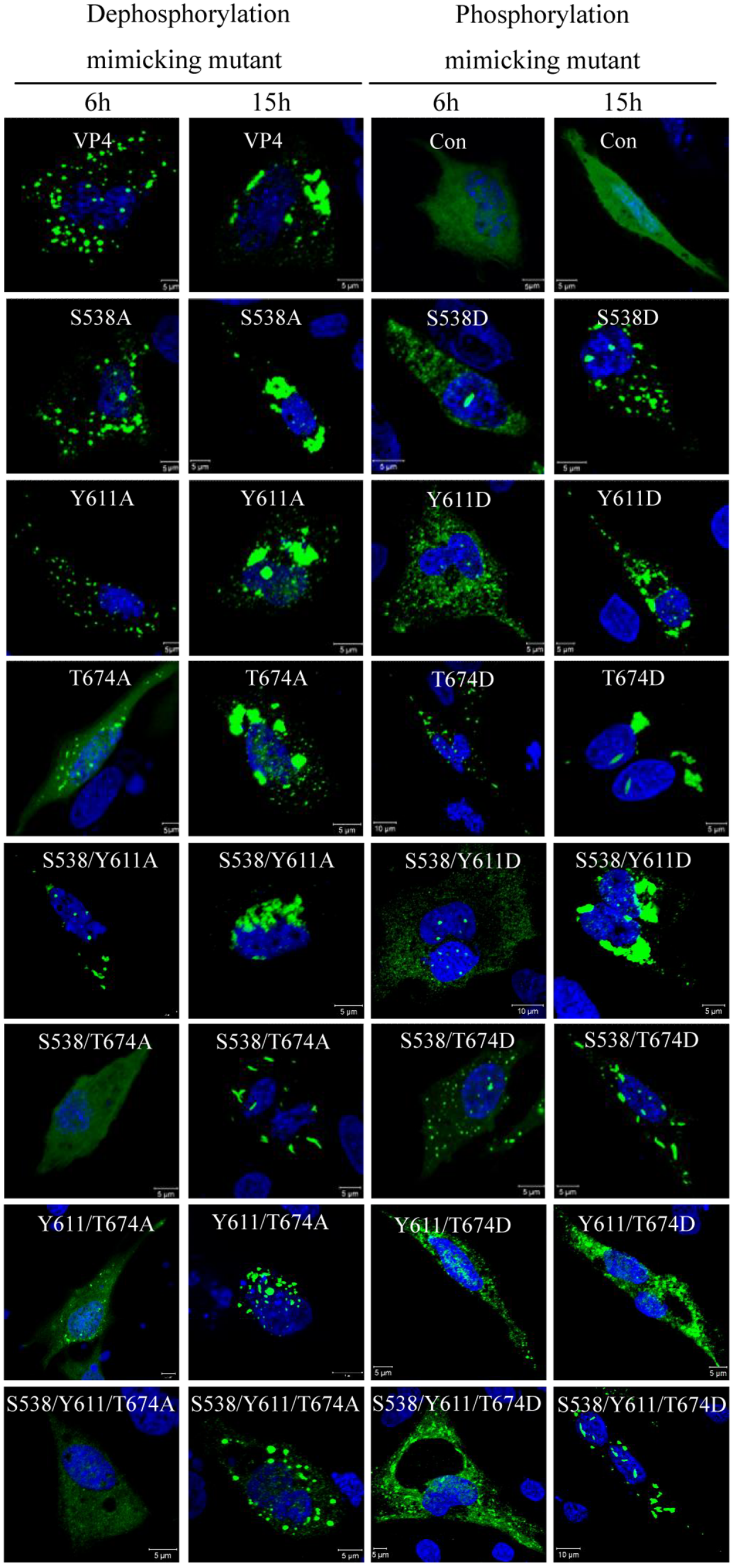
VP4 phosphorylation modifications do not affect subcellular distribution. Dephospho-mimicking (left) and phospho-mimicking (right) VP4 mutants of pSer538, pTyr611 and pThr674 were constructed by site-directed mutation of Ala or Asp substitution with the vector pEGFP-C2 and transfected into DF-1 cells. Subcellular distribution of each mutant was observed with a laser Zeiss LSM510 laser confocal microscope. Different time points post-transfection are labeled. Nuclei were counterstained with DAPI.

Substitution of phosphorylated sites with Asp (D) or Glu (E) was commonly used to mimick the phosphorylation site and study its functions [27, 29, 30]. To further analyze whether the phosphorylation modification regulates subcellular distribution, the sites Ser538, Tyr611 and Thr674 within VP4 of IBDV were mutated into Ala or Asp, and a series of dephosphorylated VP4 mutants were constructed. In cells transfected with the pEGFP-VP4 mutants (Fig 4) or pCI-VP4 mutants (S2 Fig), the expressed VP4 proteins that were mutated to Ala or Asp at the sites Ser538, Tyr611 and Thr674, aggregated into the mass-like structure of wild-type VP4 but not the rod-like or needle-like or filamentous structure VP4 in IBDV infected cells (Fig 3D). However, the VP4 aggregation is not easy to be observed at 6 h post-transfection in the VP4 mutants S538/T674A-, Y611/T674A- and S538/Y611/T674A- dephosphorylated cells (Fig 4), suggesting that the dephosphorylation of these sites potentially postpones the VP4 expression. Thus, the dephosphorylation of Ser538, Tyr611 and Thr674 did not influence the aggregation of VP4 protein, and therefore the phosphorylation of these sites may not involve the VP4 aggregation.

### The phosphorylated Tyr611 and Thr674 within VP4 protein is involved in cleavage of intermediate precursor VP4-VP3

**Fig 5:**
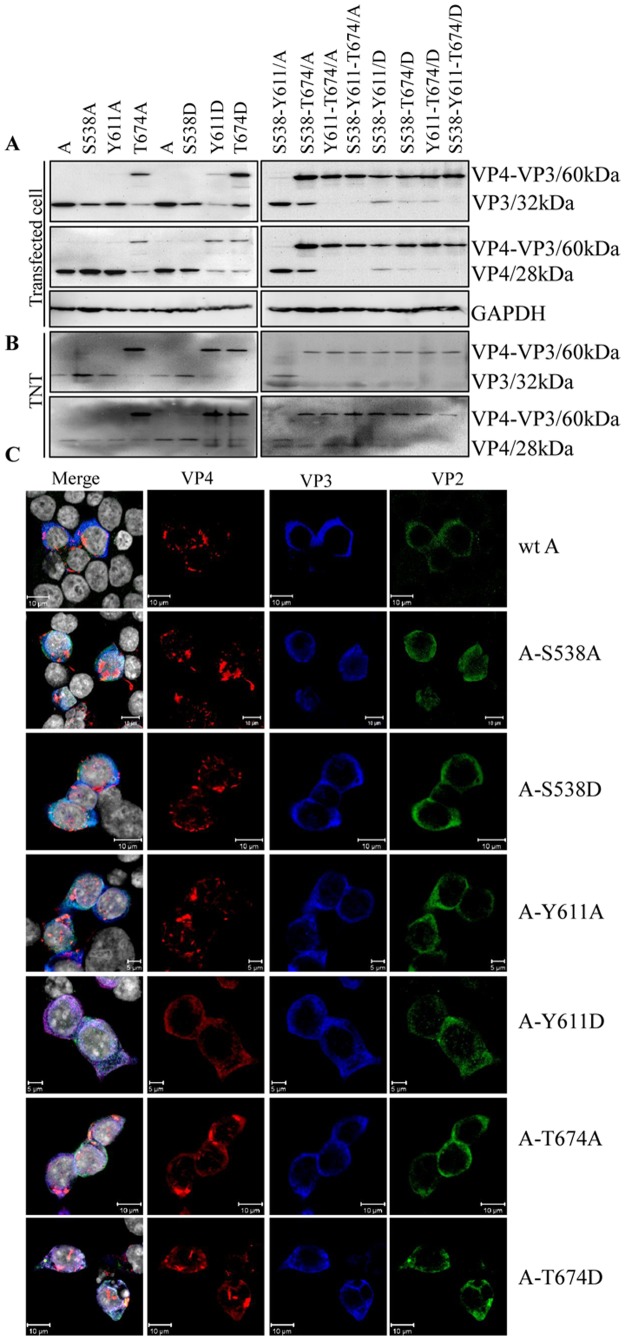
Analysis of proteolytic activity of viral VP4 protein. (A) 293T cells were transfected with the recombinant wild-type A-segment plasmid or the Ala or Asp substituted A-segment plasmids at sites pSer538, pTyr611 and pThr674 within VP4. At 24 h post-transfection, cell samples were harvested and electrophoresed on 12% SDS-PAGE gels for Western blot analysis with mAbs specific to VP3 and VP4 proteins. GAPDH was used as a loading control. (B) Recombinant plasmids used in (A) were translated with the TNT T7 Quick Coupled Transcription/Translation System, and expressed proteins were detected with mAbs specific for VP3 and VP4 proteins. (C) Analysis of co-localization between IBDV-encoding proteins within segment A. 293T cells were transfected with the IBDV A-segment mutant with the single dephospho- and phospho-mimicking VP4 gene. Wild-type IBDV A-segment transfected cells were used as a positive control. At 24 h post-transfection, the cells were fixed and probed with chicken anti-VP2 pAb, mouse anti-VP3 mAb and rabbit anti-VP4 pAb followed by FITC-conjugated goat anti-chicken IgG (green), Alexa Fluor 647 donkey anti-mouse IgG (blue) and Alexa Fluor 546 donkey ant-rabbit IgG (red). Nuclei were counterstained with DAPI (grey). The cells were observed with a laser Zeiss LSM510 laser confocal microscope. Cells transfected with the A segment with the Tyr611Asp and Thr674 Ala/Asp substitutions revealed co-localization between the IBDV-encoded proteins.

The VP4 protein of IBDV is a protease that plays an important role in the maturation of viral protein precursor. To detect whether the phosphorylation modification is involved in the proteolytic activity, various mutants of the segment A with the dephosphorylated and mimicked sites of Ser538, Tyr611 and Thr674 were constructed and transfected into cells for Western blot analysis. Only T674A/D and Y611D substitutions partially abolished the polyprotein cleavage, and the intermediate precursor VP4-VP3 protein band with a molecular weight of approximately 60 kDa was detected both with the anti-VP3 and anti-VP4 mAbs; meanwhile, substitutions at sites S538A/D and Y611A did not affect the VP4 and VP3 protein maturation (Fig 5A). Similar proteolytic activity *in vitro* was also analyzed in TNT tests. As shown in Fig 5B, any substitution at site T674 within VP4 decreased its proteolytic activity, and the intermediate precursor VP4-VP3 protein was detected. The single Ala substitution at S538 and Y611 did not affect the function of VP4 protein, while the Asp substitution of the site Y611 which mimicked Tyr phosphorylation partially affected the proteolytic activity. In further co-localization analysis (Fig 5C and S3 Fig), the signal image of VP4 and VP3 were not overlapped in the wild-type segment A of IBDV, or in segment A with the single Ala substitution at sites S538 and Y611 of VP4 protein or in segment A with the single Asp substitution at site S538 of VP4 protein. However, the co-localization of VP3 and VP4 proteins was detected in segment A with the single Ala substitution of the site T674 and in segment A with Asp-mimicked substitution of the site Y611 and T674 of VP4 protein. Taken together, these results demonstrated that the phosphorylation of Y611 and T674 maybe play an important role in the proteolytic cleavage of the intermediate precursor VP4-VP3 protein by VP4. The result of the intermediate precursor VP4-VP3 processing affected by a series of substitution was summarized in Table 4.

**Table 4:** The summary of proteolytic cleavage result affected by a series of substitution in this study.

mutation type	Ala-substitution							Asp- substitution						
position	S538	Y611	T674	S538Y611	S538T674	Y611T674	S538Y611T674	S538	Y611	T674	S538Y611	S538T674	Y611T674	S538Y611T674
result	+	+	-	+	-	-	-	+	-	-	-	-	-	-

Note:

^“+”^ means such mutation does not affect intermediate precursor VP4-VP3 cleavage;

^“-”^ means such mutation affects intermediate precursor VP4-VP3 cleavage.

## Discussion

In this report, phosphorylation of the IBDV VP4 protein at the amino acid residues Ser538, Tyr611 and Thr674 were identified by LC-MS/MS spectrum analysis (Table 3), demonstrating that the virally encoded VP4 protein is a phosphoprotein. The commercial mAbs against pSer (PSR-45, Sigma), pThr (PTR-8, Sigma) and pTyr (PT-66, Sigma) are widely used to investigate phosphorylation modifications. However, in our study these commercial antibodies failed to specifically detect the phosphorylation of Ser538, Tyr611 and Thr674 within the IBDV-encoded VP4 molecule (S4 Fig), indicating the need of developing new antibodies to detect the phosphorylated VP4. Therefore, a mAb specific for the pSer538 of the VP4 (Figs 1 and 2) was generated in this study, providing an important tool for analyzing the novel function of the phosphoprotein VP4.

The phosphorylation of viral proteins has not been reported for all members of the family Birnaviridae. In the present study, the Western blot results showed that the VP4 is abundant in all cell fractions, including the nucleus, cytoplasm, membrane and insoluble cytoskeleton, and pSer538-VP4 accounted for a small proportion of the insoluble cytoskeletal fraction. Correspondingly, in the 2-DE blots, the abundant VP4 protein and a small amount of pSer538-VP4 protein were detected in the same protein spot (Fig 3B Middle panel), as well as in two different protein spots (Fig 3B Lower panel). Further immunofluorescence analysis revealed co-localization of the VP4 proteins recognized by the pSer538 and P530 mAbs (Fig 3D). Similarly, previously published proteomic data have shown different 2-DE protein spots representing the viral VP4 protein in IBDV-infected CEF cells and bursal lymphocytes [36, 38]. These results demonstrated that the VP4 in IBDV-infected cells with pSer538 modification was a minor and insoluble protein with different isoelectric points. However, in IBDV-infected cells, why only a minor portion of VP4 protein is phosphorylated and its possible physiological meaning is unclear and needs further investigation.

Granzow *et al*. [25] reported that the intracellular type II tubule contains the IBDV VP4 protein. However, our unpublished co-localization experiments data shows there is no overlap between the cytoskeleton and mass-like, filamentous structure VP4 protein, furthermore, continuous accumulations of VP4 structures occupy a large amount of intracellular space and result in the mechanical destruction of host cytoskeletal elements (data not shown). Since collapse of the host cytoskeleton typically affects cellular integrity, this will lead to cell lysis facilitating virion egress at later stages of infection. Whether type II tubules are involved in VP4 phosphorylation is currently unknown. In the present study, the single or combined mutants mimicking dephosphorylation and phosphorylation at Ser538, Tyr611 and Thr674 of VP4, still formed a mass-like structure (Fig 4), but not a rod-like or needle-like or filamentous structure of wild-type VP4 in IBDV-infected cells (Fig 1B), indicating that these three identified phosphorylated sites within VP4 are unrelated to the formation of intracellular type II tubules. Antibody response to the IBDV VP4 protein has been reported as a biomarker discriminating the pathogenic and nonpathogenic IBDV infection [34], and VP4 protein has been reported to be an inducer of suppressing type I interferon expression via interaction with the glucocorticoid-induced leucine zipper [35]. Whether the roles are relevant to the phosphorylation of VP4 requires more in-depth investigation.

In the present study, the subcellular fractionation analysis demonstrated an abundant amount of VP4 protein in the cytoskeleton fraction (Fig 3A). However, whether there is an association between the cytoskeleton and VP4 protein is not known. The ability of a eukaryotic cell to resist deformation, to transport intracellular cargo and to change shape during movement depends on the cytoskeleton, which consists of actin filaments, microtubules and intermediate filaments, an interconnected network of filamentous polymers and regulatory proteins [42]. Many proteins interact with actin through one of the following actin-binding motifs: calponin homology domain [43], ADF-H domain [44], gelsolin homology domain [45] or thymosin β4/WH2 (WASP homology domain-2) domain, a ~35 residue actin monomer-binding motif [46]. The critical and conserved actin-binding residues are Ile, Leu and Arg/Lys all in WH2 domains [47]. Based on a publicly available service, http://elm.eu.org/, we found a potential WH2 motif within the VP4 protein of IBDV, which contains conserved marker residues ^535^Ile, ^542^Leu and ^543^Arg. Thus, it may not be surprising that the VP4 protein mainly resides in the cytoskeleton fraction (Fig 3A), and this observation may be suggestive of a potential relationship between VP4 protein and actin that is worthy of further study.

Site-directed mutagenesis studies on the VP4 protease have shown that the conserved catalytic residues serine 652 and lysine 692 in IBDV are essential for polyprotein processing [11, 48]. Similar results were also found in the VP4 protease of infectious pancreatic necrosis virus (IPNV, serine 633 and lysine 674) [49] and the blotched snakehead virus (BSNV, serine 692 and lysine 729) [50] and Tellina virus-1 (TV-1, serine 738 and lysine 777) [51]. The VP4 protease of the Birnaviridae family therefore is proposed to utilize a serine/lysine catalytic dyad mechanism to catalyze the processing of the polyprotein [11, 48, 49, 51–53]. The replacement of serine by lysine in the AXAAS motif in the VPX-VP4 boundary (^485^AQAASGTARAASGKARAAS^504^) has been found to influence polyprotein processing by VP4. Furthermore, mutation of ^514^D (^510^TLAADK^515^) was shown to prevent cleavage at the VPX-VP4 junction, while the H547P mutation abolished the polyprotein processing completely, indicating that this histidine plays a very important role in the VP4 protease catalytic activity [54]. Furthermore, the atomic structures of birnavirus VP4 proteases reveal that O^γ1^ of T712, T655 and T760 (T674 in IBDV) in the polyprotein243 of BSNV, IPNV and TV-1, is a critical donor for generating the deacylating (catalytic) water interacting with N^03B6^ of the lysine general base (Lys 729, Lys 674 and Lys 777 corresponding to BSNV, IPNV, TV-1 respectively) to form the catalytic activity domain, and the residue T674 but not Y611 in IBDV is conserved within the Birnaviridae family [51–53]. In this study, Ala or Asp substitutions (dephospho- or phospho-mimicking mutations) at the pThr674 site *in vivo* and *in vitro* both led to a marked decrease of proteolytic enzyme activity, estimating that the mutation of Thr674 abolish the ability for the lysine to function as a general base and inhibit polyprotein processing. Substitution of Asp but not Ala at pTyr611 also led to minor negative effects on proteolytic activity. Ser538 is not the active site of serine protease, therefore, Ala or Asp substitution at the pSer538 site does not affect proteolytic enzyme activity (Fig 5A and 5B). Our results above show that Tyr611 and Thr674 phosphorylation affected the maturation of intermediate precursor VP4-VP3, and further in-depth investigation about pVP2-VP4 cleavage will be followed up. In all, our results reveal that pTyr611 and pThr674 may play a partial role in the process of polyprotein cleavage.

## Supporting Information

S1 Fig Representative Coomassie stained SDS-PAGE and Western blot analysis of IBDV-infected cells. The DF-1 cells infected IBDV for 24 hour were lysed with 2-DE lysis buffer and subjected to SDS-PAGE (Left panel) and Western blot (Right panel). The anti-rabbit polyclonal antibody could react with the viral VP4 protein in IBDV-infected cells. “+”: IBDV-infected cells; “-”: mock-infected cells. The protein standard was listed in the left side.
(TIF)

S2 Fig Subcellular distribution of VP4 protein in DF-1 cells transfected with single or multiple dephospho- and phospho-mimicking VP4 mutants. Dephospho-mimicking (left) and phospho-mimicking (right) VP4 mutants of pSer538, pTyr611 and pThr674 were constructed by site-directed mutation of Ala or Asp substitution with the vector pCI-neo and transfected into DF-1 cells. Subcellular distribution of each mutant was observed with a laser Zeiss LSM510 laser confocal microscope. Different time points post-transfection are labeled. Nuclei were counterstained with DAPI.
(TIF)

S3 Fig Viral protein co-localization analysis in 293T cells transfected with IBDV segment A with the multiple dephospho- and phospho-mimicking VP4 gene. At 24 h post-transfection with the IBDV A-segment mutant with the multiple dephospho- and phospho-mimicking VP4 gene, 293T cells were fixed and probed with chicken anti-VP2 pAb, mouse anti-VP3 mAb and rabbit anti-VP4 pAb followed by FITC-conjugated goat anti-chicken IgG (green), Alexa Fluor 647 donkey anti-mouse IgG (blue) and Alexa Fluor 546 donkey ant-rabbit IgG (red). Nuclei were counterstained with DAPI (grey). The cells were observed with a laser Zeiss LSM510 laser confocal microscope. Cells transfected with the A segment with the Tyr611Asp and Thr674 Ala/Asp substitutions revealed co-localization between the IBDV-encoded proteins.
(TIF)

S4 Fig Comparison of pSer538-VP4 mAbs and general phosphor-S/T/Y mAbs. DF-1 cells infected with IBDV or not and cultured for 24 h. The cells lysed with NP-40 buffer and His-VP4 protein were subjected to SDS-PAGE and Western blot using the generated mAbs and commercial Abs. M: Protein Marker, 1: DF-1 cells infected IBDV, 2: Mock DF-1 cells, 3: Purified His-VP4. The used antibodies were shown under the picture.
(TIF)
