# Supplementary figures and images for: Avibirnavirus VP4 Protein Is a Phosphoprotein and Partially Contributes to the Cleavage of Intermediate Precursor VP4-VP3 Polyprotein
Source: PLoS One. 2015 Jun 5;10(6):e0128828. doi: 10.1371/journal.pone.0128828 (PMC4457844; doi:10.1371/journal.pone.0128828)

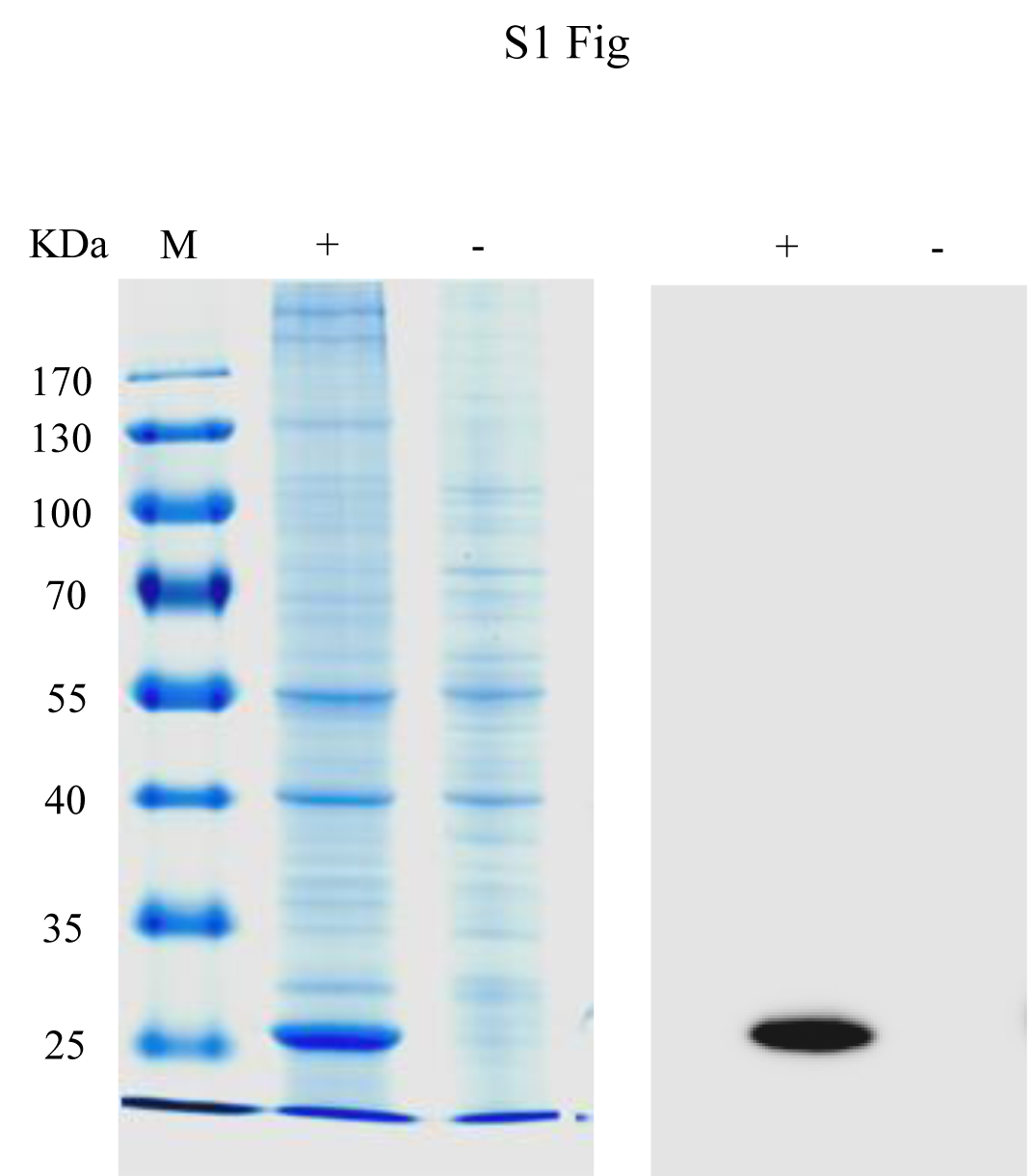

Supplement: S1 Fig — The DF-1 cells infected IBDV for 24 hour were lysed with 2-DE lysis buffer and subjected to SDS-PAGE (Left panel) and Western blot (Right panel). The anti-rabbit polyclonal antibody could react with the viral VP4 protein in IBDV-infected cells. “+”: IBDV-infected cells; “-”: mock-infected cells. The protein standard was listed in the left side. (TIF) [file pone.0128828.s001.tif]

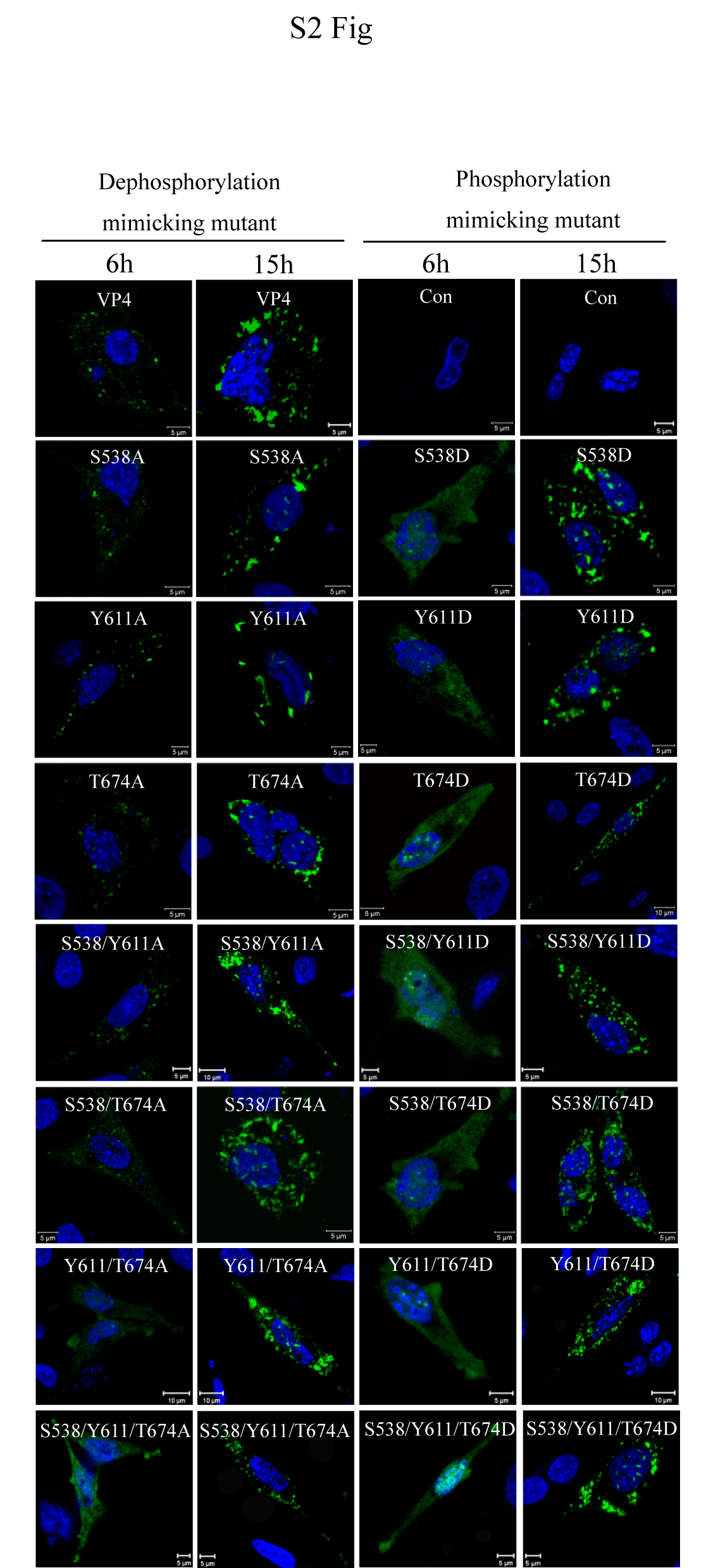

Supplement: S2 Fig — Dephospho-mimicking (left) and phospho-mimicking (right) VP4 mutants of pSer538, pTyr611 and pThr674 were constructed by site-directed mutation of Ala or Asp substitution with the vector pCI-neo and transfected into DF-1 cells. Subcellular distribution of each mutant was observed with a laser Zeiss LSM510 laser confocal microscope. Different time points post-transfection are labeled. Nuclei were counterstained with DAPI. (TIF) [file pone.0128828.s002.tif]

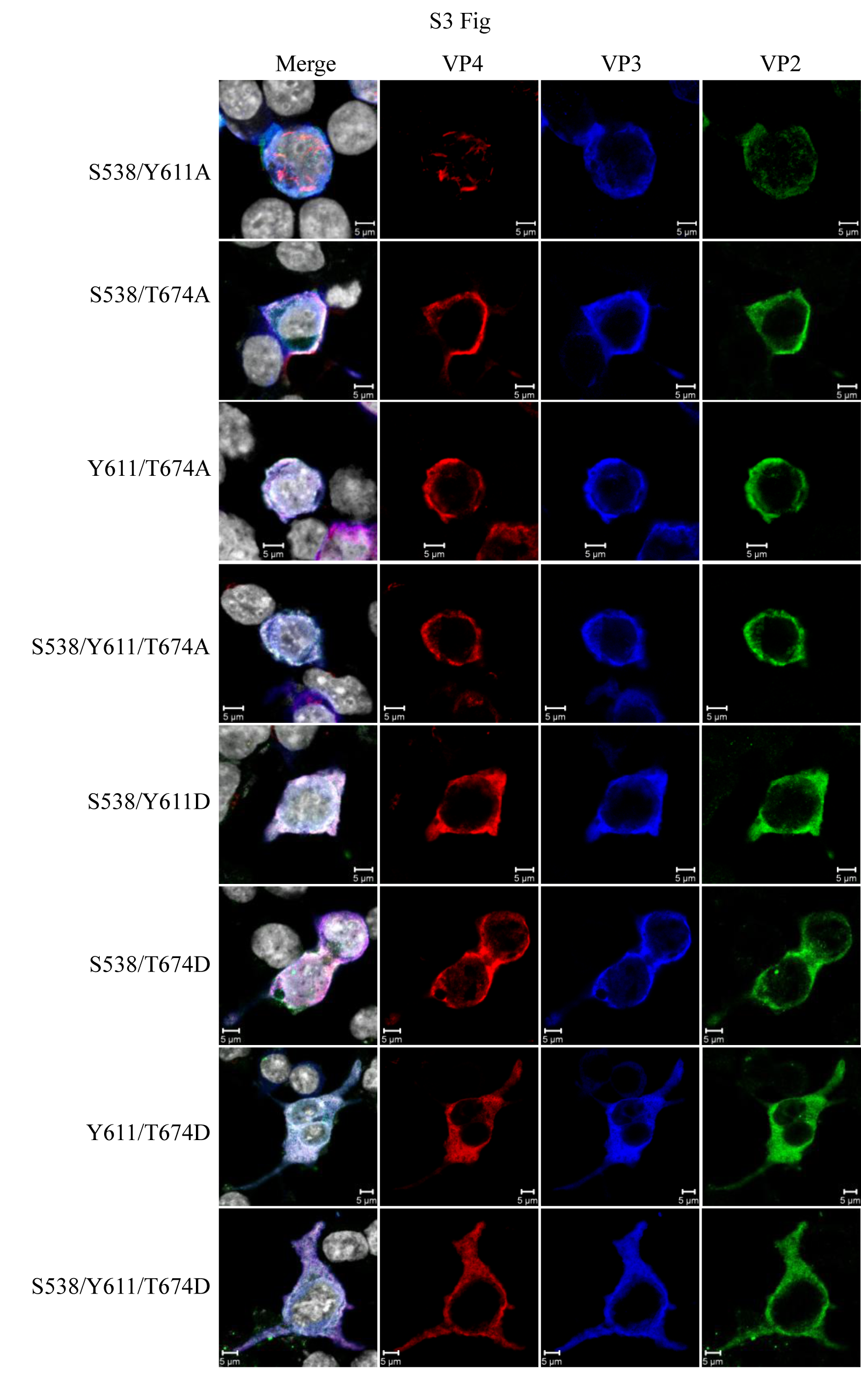

Supplement: S3 Fig — At 24 h post-transfection with the IBDV A-segment mutant with the multiple dephospho- and phospho-mimicking VP4 gene, 293T cells were fixed and probed with chicken anti-VP2 pAb, mouse anti-VP3 mAb and rabbit anti-VP4 pAb followed by FITC-conjugated goat anti-chicken IgG (green), Alexa Fluor 647 donkey anti-mouse IgG (blue) and Alexa Fluor 546 donkey ant-rabbit IgG (red). Nuclei were counterstained with DAPI (grey). The cells were observed with a laser Zeiss LSM510 laser confocal microscope. Cells transfected with the A segment with the Tyr611Asp and Thr674 Ala/Asp substitutions revealed co-localization between the IBDV-encoded proteins. (TIF) [file pone.0128828.s003.tif]

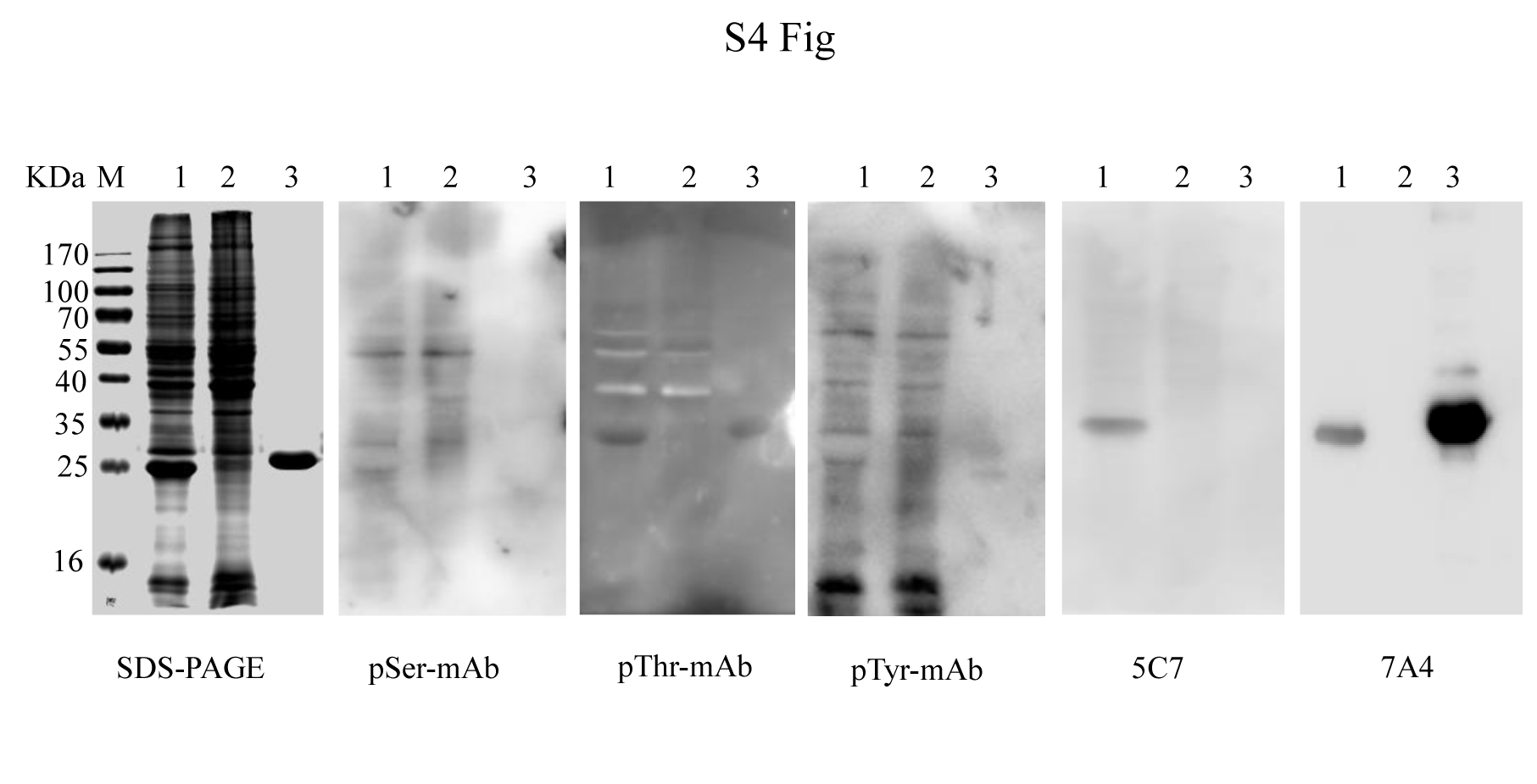

Supplement: S4 Fig — DF-1 cells infected with IBDV or not and cultured for 24 h. The cells lysed with NP-40 buffer and His-VP4 protein were subjected to SDS-PAGE and Western blot using the generated mAbs and commercial Abs. M: Protein Marker, 1: DF-1 cells infected IBDV, 2: Mock DF-1 cells, 3: Purified His-VP4. The used antibodies were shown under the picture. (TIF) [file pone.0128828.s004.tif]
